# Effect of one time high dose “stoss therapy” of vitamin D on glucose homeostasis in high risk obese adolescents

**DOI:** 10.20945/2359-3997000000024

**Published:** 2018-03-23

**Authors:** Preneet Cheema Brar, Maria Contreras, Xiaozhou Fan, Nipapat Visavachaipan

**Affiliations:** 1 New York University School of Medicine Division of Pediatric Endocrinology Department of Pediatrics New York USA Department of Pediatrics, Division of Pediatric Endocrinology, New York University School of Medicine, New York, USA; 2 Texas Tech University Health Science Center Department of Pediatrics Amarillo Texas USA Texas Tech University Health Science Center, Department of Pediatrics, Amarillo, Texas, USA; 3 New York University School of Medicine Department of Population Health New York USA Department of Population Health, New York University School of Medicine, New York, USA; 4 Bumrungrad International Hospital Bangkok Thailand Bumrungrad International Hospital, Bangkok, Thailand

**Keywords:** Vitamin D, insulin resistance, prediabetes, obesity

## Abstract

**Objective:**

To study the effect of using a one time high dose “stoss therapy” of vitamin D2 (ergocalciferol: VD2) on indices of insulin sensitivity {whole body sensitivity index: WBISI} and secretion {insulinogenic index: IGI} measured during an oral glucose tolerance test (OGTT) in obese adolescents with VDD (25 OHD; serum metabolite of vit D: < 30 ng/dL).

**Subjects and methods:**

In a randomized placebo controlled cross over design 20 obese adolescents with vitamin D deficiency (VDD) had baseline OGTT. Arm A received one time high dose 300,000 IU of ergocalciferol and Arm B received placebo. After 6 weeks the adolescents were reassigned to Arm A if they were in Arm B and vice versa. 25OHD, calcium, parathyroid hormone, comprehensive metabolic panel, urine calcium creatinine ratio were measured at each study visit. OGTTs to assess indices of sensitivity and secretion were done at baseline, 6 weeks and 12 weeks respectively.

**Results:**

Adolescents were obese and insulin resistant (mean ± SD: mean age = 15.1 ± 1.9 years; BMI: 32.7 ± 9.8; homeostatic model of insulin resistance: HOMA-IR: 4.2 ± 2.8). Stoss therapy with VD2 increased 25OHD from baseline (16.7 ± 2.9 to 19.5 ± 4.5; p = 0.0029) when compared to the placebo. WBISI (2.8 ± 1.9) showed a trend towards improvement in Rx group (p = 0.0577) after adjustment for covariates. IGI (3 ± 2.2) showed an improvement in both Rx and placebo groups.

**Conclusions:**

Our study demonstrated that using a high dose of VD2 (300,000 IU) did not have any beneficial effect on insulin sensitivity (whole body sensitivity index {WBISI}) and secretory indices (insulinogenic index {IGI}) in obese adolescents. High dose “stoss therapy” of VD2 did not appear to have any beneficial effect on glucose homeostasis on obese adolescents.

## INTRODUCTION

Low vitamin D levels are consistently seen in 32-50% of obese adolescents (1–3). It is also thought that these low levels could be due to differences in vitamin D metabolizing enzymes in adipose tissue (4,5) and higher volumetric dilution of serum vitamin D, rather than just sequestration in adipose tissue, which could explain these lower levels of vitamin D in obese adolescents when compared to their lean peers.

Vitamin D has been shown in both in vivo and in vitro studies to have effects on beta cell function and insulin sensitivity (6,7). The role of vitamin D in glucose homeostasis is well established and prospective studies have shown that vitamin D deficiency has an inverse and significant association with prediabetes and/or Type 2 diabetes (8,9).

There have been inconsistent results in randomized controlled trials done to study the effect of vitamin D supplementation on parameters of glucose homeostasis in insulin resistance states, in both adults and children, with some showing beneficial effects on insulin sensitivity (10–13) while others did not (14). In adults, the effect of vitamin D on prediabetes and/or T2DM showed beneficial effect in a study by Neyestani and cols. (15) and no effect in another (16). More recently studies using high dosing vitamin D (150,000-300,000 units) over a short duration (4-8 weeks) have also shown conflicting results on insulin sensitivity and secretion in adults with prediabetes (17,18).

There has been no RCT which has been done in obese adolescents with vitamin D deficiency, defined as a 25(OH) D level of < 30 ng/dL (75 nmol/L) (19) and insulin resistance to assess the efficacy of using one time high dose of VD2 on indices of insulin sensitivity and secretion over a short period of time. To summarize we tested whether one time high dose of ergocalciferol (300,000 units) corrected the vitamin D deficiency and improved glucose homeostasis in obese adolescents with insulin resistance.

## SUBJECTS AND METHODS

This was a randomized placebo controlled cross over design trial with inclusion criteria that were: a) obese adolescents (BMI: ≥ 95^th^ percentile for age) who were 12-18 years; b) > Tanner 2 for puberty and had vitamin D deficiency defined as a 25(OH)D of ≤ 20 ng/mL (50 nmol/L). Exclusion criteria were: a) treatment with medication known to effect vitamin D, calcium and glucose metabolism, such as glucocorticoids, thiazolidinediones, metformin, anticonvulsants metabolized through cytochrome P-450 (phenytoin, carbamazepine, phenobarbital, sodium valproate); b) vitamin D supplementation greater than 400 IU daily in the preceding 3 months; c) history of nephrolithiasis or hypercalcemia; pregnancy; d) attendance at a tanning salon. The study was approved by the Ethics committee at New York University School of Medicine and consent was obtained from parents and patients.

We chose to use “stoss” therapy {German word stossen means “to push”} based on a recent global consensus for management of vitamin D deficiency (20). The Endocrine society consensus statement recommended 50,000 for 6 weeks for children and adolescents with vitamin D deficiency. Much higher dosing was recommended for obese adults at least 6,000-10,000 per day for 8 weeks (19). We decided to give 300,000 IU as a “stoss dose”, a practical choice to improve compliance. Subjects selected for the study were randomized, half to the treatment group (A) and half to the placebo group (B). At week 7, subjects were switched over and reassigned to receive vitamin D if they are in Group B and placebo if they were in Group A and the study lasted 12 weeks from start to completion.

**Figure 1:**
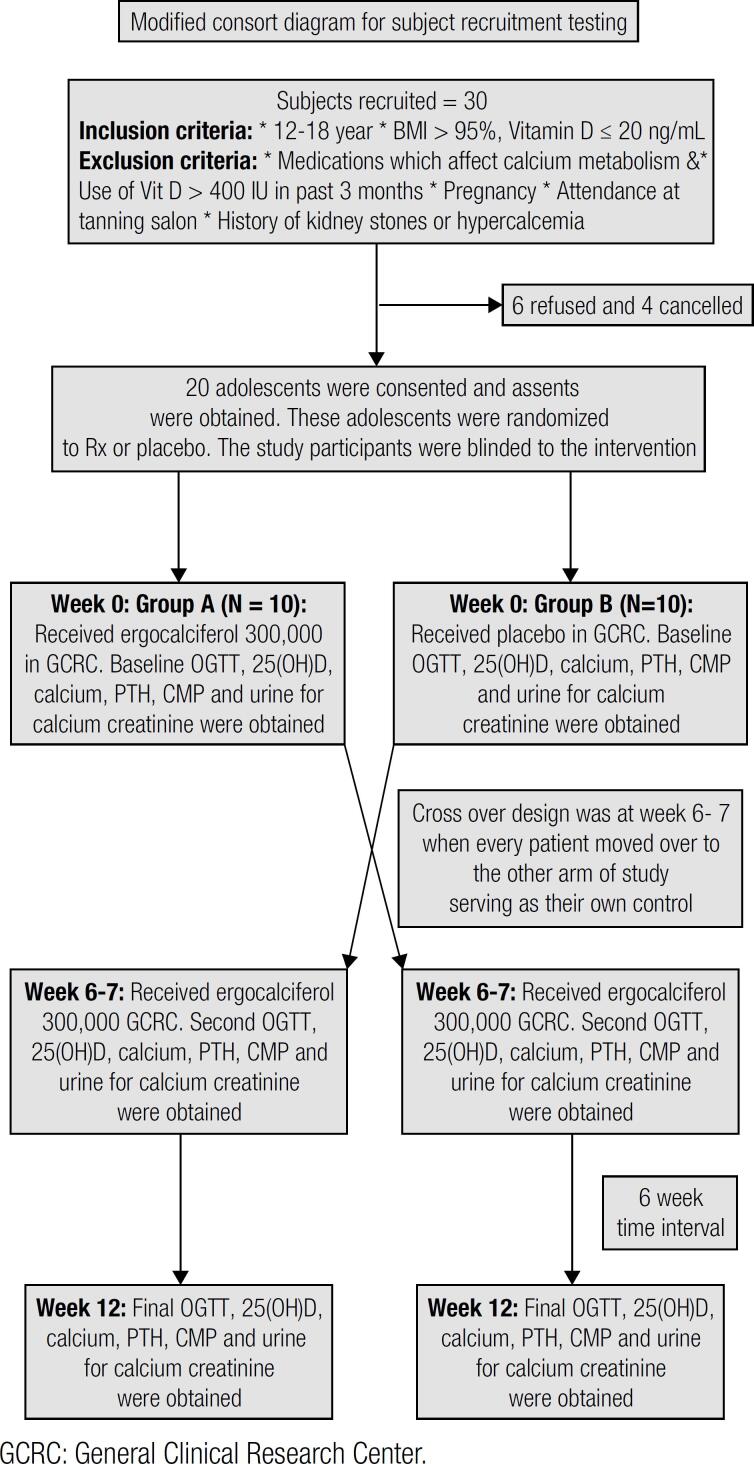
Consort diagram to show recruitment and study design.

Ergocalciferol (50,000 IU) capsules and placebo capsules were provided at the study visit based on the randomization scheme. Each subject got 6 capsules of study drug or placebo at the study visit totaling 300,000 IU of ergocalciferol or no ergocalciferol at all in the placebo capsules. Each arm of trial lasted 6 weeks with no washout period. Patients were blinded to treatment assignment during the entire study. Study design and recruitment are shown in the study consort diagram (Figure 1).

The study subjects had an oral glucose tolerance test with 75 g of glucose solution (OGTT) and screening labs were drawn at baseline, at week 7 and then again week 12 at the completion of the study. Plasma glucose and insulin will were measured using Luminex technology. Serum 25-OH vitamin D were measured using liquid chromatography, tandem mass spectrometry (LC/MS/MS), which consists of extraction via protein precipitation, separation via highperformance liquid chromatography (HPLC), detection and quantitation via tandem mass spectrometry. 25OHD_2_ and 25OHD_3_ concentrations were used to calculate total 25OHD levels. Glycosylated hemoglobin (HbA1C) were measured in red blood cells using HPLC method. Serum calcium (mg/dL), albumin (g/dL) and intact parathyroid hormone (PTH) (pg/mL were measured. Calcium was corrected for the serum albumin {([4-albumin (g/dL)] x 0.8) + calcium (mg/dL)} (21). Intact PTH, 25(OH) D and spot urine calcium/creatinine ratio were checked at completion of the 6 week treatment phase (in week 7) to exclude vitamin D toxicity including hypercalciuria (urine calcium/creatinine ratio ≥ 0.2), hyperphosphatemia (serum phosphate > 5.7 mg/mL), hypercalcemia (serum calcium > 10.5 mg/dL), serum 25(OH) D > 150 ng/mL. Baseline labs were drawn at the same time as 0 minute OGTT.

### Primary outcome

OGTT was done glucose solution (1.75 g/kg up to a maximum of 75 g) over a 2-minute period and blood samples were obtained at 0, 10, 30, 60, 90 and 120 minutes. Indices were calculated from OGTTs done at three time points: baseline; 7 week and 12 week time points.

#### Calculated insulin sensitivity parameter from OGTT

Whole body insulin sensitivity (WBISI) (22) is an insulin sensitivity measure that has been validated in obese children and adolescents (23) calculated as follows = 10,000/√(fasting glucose mg/dl × fasting insulinµIU/ml)×(mean glucose × mean insulin) during OGTT 1 during OGTT. Higher WBISI levels indicate greater insulin sensitivity.

#### Calculated insulin secretory parameters from OGTT

Insulin index (IGI): is a measure of insulin secretion that has been validated in children against the hyperglycemic clamp [7], calculated as followed: IGI = [30-minute insulin – fasting plasma insulin (uIU/mL)/[30-minute glucose – fasting plasma glucose (mg/dL)]. Adolescents with Type 2 diabetes have a significant reduction in IGI (24).

#### Secondary outcome

a. Pre- and post treatment 25 OHD; b. change in serum PTH; c. Biochemical evidence of vitamin D toxicity such hypercalciuria (urine calcium/creatinine ratio ≥ 0.2), serum phosphate > 5.7 mg/mL, serum calcium > 10.5 mg/dL, serum 25(OH) D > 150 ng/mL.

### Statistical analysis

To estimate the effect of vitamin D treatment on the clinical features in this crossover study, we first examined the within patient comparison by using paired t-test. We next examined the treatment given one period (treatment/placebo) adjusted for the baseline values by using mixed regression models controlling for baseline measurements, age, gender, race, BMI, and seasons [winter: Dec-Feb; spring: Mar-May; summer: Jun-Aug; fall: Sep-Nov]. Koch's test was used to examine the crossover effect on the association of treatment and clinical features. We further conducted stratified analyses according to baseline diabetes and pre-diabetes status, using the criteria plasma glucose at 0 minute ≥ 100 mg/dL or at 120 minutes ≥ 140 in oral glucose tolerance test (OGTT), HbA1C ≥ 5.7% and HOMA -IR ≥ 3.4, and intact PTH ≥ 44 pg/mL. At last, plasma glucose concentration and insulin level at times 0, minutes, 30 minutes, 60 minutes, 90 minutes, and 120 minutes in insulin sensitivity test were compared between treatment and placebo groups by using the mixed regression models described above, as well as the mean levels at the first-phase (at and before 30 minutes), second phase (after 30 minutes), and whole period (0-120 minutes). All statistical tests were two-sided, and all statistical analyses were carried out using SAS 9.3.

## RESULTS

Of the twenty participants, 80% (n = 16) were females, and 75% (n = 15) were Hispanic, with mean age 15 year-old. The study participants were predominantly overweight, with mean BMI 32.7 (Table 1). The baseline clinical features of all participants were shown in Table 1 as well: Serum 25-OH vitamin D levels were 16.7 ± 2.9 ng/mL (reference range 12-20 ng/mL) (25); and WBISI were 2.8 ± 1.9 (reference range 1.84 ± 0.17); and IGI were 3.0 ± 2.2; and PTH were 50.9 ± 15.8 (reference range 15 – 75 pg/mL).

**Table 1:** Baseline characteristics and clinical features of the study population

Characteristics and clinical features		Study participants (n = 20)	
		Mean/N	SD/%
Age (year)		15.1	1.9
Gender*			
	Male	4	20
	Female	16	80
Ethnicity*			
	African American	3	15
	Bangladesh	1	5
	Caucasian	1	5
	Hispanic	15	75
Height (cm)		162.5	7.8
Weight (kg)		90.7	19.2
BMI		32.7	9.8
25 OH vitamin D (ng/mL)		16.7	2.9
WBISI		2.8	1.9
IGI		3.0	2.2
Intact, PTH (pg/mL)		50.9	15.8
HbA1C		5.7	0.3
HOMA-B%		452.2	343.3
HOMA-IR		4.2	2.8
Serum calcium (mg/dL)		9.4	0.4
Albumin (g/dL)		4.2	0.3
Albumin-adjusted serum calcium (mg/dL)		9.2	0.4
Alkaline phosphatase (U/L)		110.0	46.8
AST (U/L)		29.2	14.1
ALT (U/L)		42.3	39.8
Serum phosphorus (mg/dL)		4.5	0.6
Random urine calcium (mg/dL)		6.5	4.6
Random urine creatinine (mg/dL)		217.9	107.2

* Numbers of participants and percentage were calculated.

We first examined the effect of vitamin D treatment on the serum 25-OH vitamin D levels (Table 2). Treatment group had significant increased serum 25-OH vitamin D levels (19.5 ± 4.5 ng/mL; p from paired t-test = 0.0029) compared to baseline levels. This increase in serum 25-OH vitamin D levels after treatment was significantly different relative to placebo group, after further adjusted for covariates (adjusted p from mixed model = 0.0059), and did not due to crossover effect (p from Koch's analysis = 0.4506).

**Table 2:** Baseline values and changes after treatment in clinical features and the placebo group

	Baseline		Treatment group (N = 20)					Placebo group (N = 20)						
			∆ after treatment					∆ after treatment						
	Mean	SD	Mean	SD	Median*	IQR*	p†	Mean	SD	Median*	IQR*	p†	p‡	p§
25 OH vitamin D (ng/mL)	16.7	2.9	19.5	4.5	2.4	4.7	0.0029	17.2	4.7	0.0	2.2	0.5262	0.0059	0.4506
WBISI	2.8	1.9	2.7	1.4	-0.1	1.1	0.5377	3.1	1.5	0.3	1.9	0.3855	0.0577	0.4205
IGI	3.0	2.2	3.5	2.7	0.1	1.8	0.2878	4.3	6.3	0.1	1.2	0.3069	0.5971	0.0030
Intact, PTH (pg/mL)	50.9	15.8	45.6	11.8	-6.0	19.0	0.0538	50.3	14.3	0.5	16.0	0.8327	0.1290	0.1075
HbA1C	5.7	0.3	5.7	0.4	0.1	0.2	0.2674	5.7	0.4	0.1	0.3	0.3264	0.9052	0.2918
HOMA-B%	452.2	343.3	483.5	258.2	19.8	173.8	0.3889	543.9	532.2	34.2	288.5	0.2769	0.5740	0.0004
HOMA-IR	4.2	2.8	5.2	3.6	0.9	2.3	0.1079	4.9	4.5	0.2	1.3	0.2829	0.3403	0.3396
Serum calcium (mg/dL)	9.4	0.4	9.3	0.4	0.0	0.4	0.4936	9.2	0.3	-0.1	0.5	0.0909	0.1287	0.2258
Albumin (g/dL)	4.2	0.3	4.2	0.9	-0.3	0.4	0.8346	4.1	0.3	-0.2	0.3	0.1670	0.8932	0.0200
Albumin-adjusted serum calcium	9.2	0.4	9.1	0.7	0.1	0.4	0.8927	14.7	25.6	0.0	0.6	0.3750	0.3975	< 0.0001
Alkaline phosphatase (U/L)	110.0	46.8	103.6	43.5	-6.5	13.0	0.0344	99.6	37.6	-7.5	15.0	0.0034	0.3105	0.4072
AST (U/L)	29.2	14.1	25.2	11.9	-2.0	5.5	0.0209	29.1	15.8	-0.5	6.0	0.9374	0.0870	< 0.0001
ALT (U/L)	42.3	39.8	36.2	32.4	-4.0	9.5	0.0106	40.6	36.7	-1.0	9.0	0.6514	0.2704	< 0.0001
Serum phosphorus (mg/dL)	4.5	0.6	4.5	0.6	-0.1	0.5	0.7354	4.5	0.5	0.0	0.4	0.5965	0.6586	0.9649
Random urine calcium (mg/dL)	6.5	4.6	8.6	7.8	2.7	6.2	0.1733	14.0	21.4	1.0	7.0	0.1411	0.3369	0.0007
Random urine creatinine (mg/dL)	217.9	107.2	274.6	441.8	-12.7	106.5	0.5034	199.2	108.3	-9.9	89.7	0.4150	0.3292	< 0.0001

* Medians and IQR of the difference between treatment/placebo group and baseline were calculated.

† p values were calculated from exact comparison (paired t-test) of measurements to baseline.

‡ p values were calculated from mixed regression models, adjusted for baseline measurements, age, gender, race, BMI, and season [winter: Dec-Feb; spring: Mar-May; summer: Jun-Aug; fall: Sep-Nov].

§ p values were calculated from Koch's analysis testing for crossover effects.

We next examined that if the vitamin D treatment were associated with insulin sensitivity and secretory parameters (Table 2). The means of whole body insulin sensitivity (WBISI) tend to be increased (**∆**_mean_ = 0.1, p_t-test_ = 0.5377) in treatment group and decreased (**∆**_mean_ = 0.3, p_t-test_ = 0.3855) in placebo group compared to baseline levels, however, without statistical significance. Whereas, the difference in WBISI between treatment and placebo group was marginally significant after adjusted for covariates (adjusted p from mixed model = 0.0577). The means of insulin index (IGI) increased with 0.1 in both treatment and placebo group compared to baseline, and these differences were not statistically significant. Intact PTH decreased in treatment group (**∆**_mean_ = -6.0, p_t-test_ = 0.0538); however, this decrease was not significant compared to the changes in placebo group (adjusted p from mixed model = 0.1290). Additionally, alkaline phosphatase levels decreased in both treatment group (**∆**_mean_ = -6.5, p_t-test_ = 0.0334) and placebo group (**∆**_mean_ = -7.5, p_t-test_ = 0.0034), and AST and ALT only decreased in treatment group (**∆**_mean_ = -2.0, p_t-test_ = 0.0209 for AST, and **∆**_mean_ = -4.0, p_t-test_ = 0.0106 for ALT). However, the decreasewere not significant compared to the changes in placebo group.

Since childhood diabetes and pre-diabetes status may have effects of vitamin D treatment on insulin sensitivity, we stratified the associations of VD2 treatment with WBISI and IGI by clinical diabetes measurements (Table 3). In contrast to the overall analysis, the means of IGI decreased in treatment (**∆**_mean_ = -0.70, p_t-test_ = 0.5606) in children with fasting serum glucose level ≥ 100 or ≥ 140 at time 0 and 120 minutes during the OGTT, although these differences were not significant. Children with HOMA-IR ≥ 3.4 and HbA1C ≥ 5.7% had similar trend of in both treatment and placebo group with the overall changes. When stratified by PTH, the treatment group with PTH ≤ 44 pg/mL had increased mean of WBISI (**∆**_mean_ = 0.10, p_ttest_ = 0.5040), and decreased mean of IGI (**∆**_mean_ = -0.30, p_t-test_ = 0.5722).

**Table 3:** Changes after treatment in WBISI and IGI and the placebo group stratified according to diabetes status

	Treatment group						Placebo group							
	∆ after treatment						∆ after treatment							
	N	Mean	SD	Median*	IQR*	p^†^	N	Mean	SD	Median*	IQR*	p^†^	p^‡^	p^§^
G_0 ≥ 100 mg/dL and/ or G_120 ≥ 140 mg/dL	5						5							
WBISI		1.38	0.80	-0.20	0.30	0.1293		1.56	1.04	-0.10	0.50	0.6213	0.4718	0.5477
IGI		5.18	4.51	-0.70	1.20	0.5606		2.94	2.43	-0.60	1.60	0.1376	0.1236	0.1126
HOMA-IR ≥ 3.4 & HbA1C ≥ 5.7%	6						6							
WBISI		2.27	1.44	-0.25	0.30	0.8226		2.32	1.06	0.25	0.90	0.4391	0.2516	0.1764
IGI		5.70	3.52	2.30	5.60	0.2714		8.40	10.69	0.95	5.60	0.2636	0.1735	0.8965
Intact, PTH ≤ 44 pg/mL	9						9							
WBISI		2.15	1.18	0.10	0.35	0.5040		2.86	1.69	0.30	2.00	0.0765	0.3445	< 0.0001
IGI		3.26	2.20	-0.30	1.10	0.5722		2.64	1.88	-0.55	1.40	0.0389	0.3450	< 0.0001
Intact, PTH > 44 pg/mL	11						11							
WBISI		3.05	1.43	-0.30	1.90	0.4458		3.34	1.46	0.20	1.60	0.7871	0.2312	0.4084
IGI		3.74	3.20	0.65	3.20	0.2286		5.61	8.24	0.50	1.50	0.1978	0.7405	0.1898

N: number; SD: standard deviation; IQR: inter quartile range; p: *ρ* value.

In the comparisons of the mean differences between treatment and placebo group at each time point of OGTT (Table 4: 0 min, 10 min, 30 min, 60 min, 90 min, 120 min), serum glucose level decreased slower in treatment group (**∆**_mean_ = -5.5, p_t-test_ = 0.2162) than placebo (**∆**_mean_ = -18.5, p_t-test_ = 0.0007) at 30 minutes, with p value from mixed model equals to 0.0236. While, insulin level increased in treatment group (**∆**_mean_ = 9.0, p_t-test_ = 0. 7864), and decreased in placebo (**∆**_mean_ = -6.0, p_t-test_ = 0.1270) at 30 minutes (p from mixed model = 0.0197). No significant differences between treatment and placebo at other time point of OGTT was found, as well as the mean values of all time points in both glucose and insulin levels.

**Table 4:** Baseline values and changes after treatment in plasma glucose and insulin levels and the placebo group at each time point

		Baseline		Treatment group (N = 20)					Placebo (N = 20)						
				∆ after treatment					∆ after treatment						
		Mean	SD	Mean	SD	Mean*	SD*	p†	Mean	SD	Mean*	SD*	p†	p‡	p§
Plasma glucose															
	At fasting	82.0	7.2	82.0	5.6	0.0	8.5	0.9698	81.2	5.5	-0.5	7.0	0.4466	0.3645	0.2856
	At 10 min	102.4	16.1	100.6	13.3	1.5	9.5	0.5910	96.7	13.5	-2.5	12.0	0.0589	0.2423	0.1161
	At 30 min	139.8	25.4	133.4	22.6	-5.5	33.0	0.2162	122.5	22.1	-18.5	25.0	0.0007	0.0236	0.4265
	At 60 min	139.8	28.5	128.7	38.9	-3.5	55.5	0.1202	119.8	34.0	-21.0	37.0	0.0067	0.2243	0.0452
	At 90 min	127.5	29.4	123.4	34.9	-10.5	53.0	0.5524	117.7	39.9	-10.5	29.0	0.2737	0.4540	0.1342
	At 120 min	123.3	31.2	110.5	31.0	-13.5	28.5	0.0568	111.8	35.1	-11.0	19.0	0.0419	0.9691	0.0700
	Mean	119.1	18.4	113.1	20.1	-6.9	27.0	0.0684	108.3	22.0	-10.7	19.4	0.0037	0.1754	0.1741
Insulin															
	At fasting	20.9	13.3	25.2	16.7	4.0	10.0	0.0749	24.1	21.7	0.5	6.5	0.2686	0.4555	0.7516
	At 10 min	77.7	63.4	74.2	49.5	5.0	27.0	0.7478	71.9	61.7	3.5	52.0	0.6103	0.8206	0.6579
	At 30 min	183.5	147.6	176.9	110.1	9.0	117.0	0.7864	151.4	116.7	-6.0	108.5	0.1270	0.0197	0.3541
	At 60 min	213.9	183.6	187.0	181.3	-8.0	95.0	0.2746	145.8	136.3	-41.5	105.5	0.0178	0.1340	0.3023
	At 90 min	207.6	253.6	204.7	253.6	-17.5	133.0	0.3248	148.0	185.0	-58.0	117.0	0.0245	0.0604	0.0242
	At 120 min	231.3	352.4	138.7	116.3	-37.0	109.5	0.1919	165.7	250.4	-31.0	80.5	0.0487	0.6277	0.0135
	Mean	156.0	158.9	136.0	115.7	-5.5	54.4	0.2027	117.8	121.6	-2970.0	59.8	0.0215	0.2580	0.1541
	First-phase (≤ 30 m)	49.3	35.3	52.1	34.9	5.5	17.8	0.6396	48.0	40.3	0.8	33.0	0.8336	0.5587	0.5024
	Second-phase (> 30 m)	211.8	225.3	175.4	153.8	-20.3	83.3	0.1284	152.7	164.2	-36.8	87.0	0.0167	0.2367	0.3730

* Medians and IQR of the difference between treatment/placebo group and baseline were calculated.

† p values were calculated from exact comparison (paired t-test) of measurements to baseline.

‡ p values were calculated from mixed regression models, adjusted for baseline measurements, age, gender, race, BMI, and season [winter: Dec-Feb; spring: Mar-May; summer: Jun-Aug; fall: Sep-Nov].

§ p values were calculated from Koch's analysis testing for crossover effects.

## DISCUSSION

Our study demonstrated that using high dose of VD2 (300,000 IU) in a cross over design trial did not have any beneficial effect on insulin sensitivity and secretory indices in obese adolescents when measured using an oral glucose tolerance test.

Prediabetes was found in 10-39% of obese adolescents (24,26), which parallels the rise of obesity (27). Vitamin D has shown to have effects on insulin secretion and action and in both pediatric and adult studies an inverse association between vitamin D levels and development of prediabetes and/or T2DM have been demonstrated (10,21).

Long term randomized control trials (3 months-7 years) have studied whether giving vitamin D prevents the progression of insulin resistance to prediabetes to Type 2 diabetes due to its effects on augmenting insulin action and secretion. Von Hurst and cols. studied 42 South Asian women with insulin resistance using 4000 IU of D3 for 6 months. Fasting insulin and HOMA-IR improved in the cases versus controls (p values = 0.02). Davidson and cols. used a weight and vitamin D based formula to calculate vitamin D dosing (average of 88000 IU/week) for 12 months and showed improvement in Hba1c (decrease by 0.2%) but the intervention affected no other parameters of any OGTT derived secretory and sensitivity indices in this cohort of pre diabetic adults. These studies (more than 6 months of vitamin D treatment) have been equivocal to truly establish any real benefit of using vitamin D on indices of beta cell function and insulin sensitivity, the caveat being the variations in the dosing of vitamin D used, compliance concerns and whether the vitamin D truly reached an optimal level (i.e. > 30 ng/mL) to effect the aspects of function of beta cell. It is clear that in this obese cohort of female adolescents (average BMI = 32.1) we found that treatment did increase the vitamin D level when compared to the placebo arm (19.5 vs. 17.2 ng/dL; p = 0.0029) and this significance stayed after adjustments for covariates: BMI, age sex, gender, race and season. However our intervention of 300,000 IU was not able to optimize the vitamin D levels to levels of sufficiency i.e. ≥ 30 ng/mL in all except one subjects. Levels reached ≥ 20 ng/mL in six subjects by the end of the three month intervention. This dosing was based on the Endocrine society guidelines of using 50,000 IU for 6 weeks for deficient states (19).

We wanted to test the effect of using high dose of vitamin D effect on insulin sensitivity and secretory indices. WBISI did show an increase in the Rx group (delta mean increase 0.1) though this difference was not significant when compared to the placebo. IGI increased in both groups while PTH decreased to a greater extent when compared to the placebo though in the mixed model analysis the difference was not significant. In a similar study designed by Ashraf and cols. obese adolescents (average age 14.9 ± 1.8 years) were given 50,000 IU of vitamin D per week for 8 weeks to observe effects on glucose parameters. While HOMA-IR and WBISI did not improve on the follow up OGTT fasting glucose showed statistical improvement (p = 0.05) in cases when compared to controls. In a dose titration study (400 IU to 4000 IU) of 323 early pubertal children (age = 11.3 years; BMI% 70% and 25 (OH) levels 28 ng/mL) fasting insulin and HOMA-IR correlated with baseline levels of 25(OH) D (r = 0.14 and 0.15 respectively). Rx with vitamin D had no significant positive impact on glucose and insulin parameters over a 12 week period. In this study by Ferira and cols. among these children only 15% were vitamin D deficient and obese respectively and therefore making comparisons with our study results would not be reasonable (28).

In a noteworthy study by Wagner and cols. investigated the effect of high-dose vitamin D3 treatment on beta-cell function, insulin sensitivity, and glucose tolerance in subjects with prediabetes or diet-treated type 2 diabetes adults (n = 43, BMI 28.6) randomized to 30,000 IU of cholecalciferol or placebo drops weekly for 8 weeks. They studied first and second phase insulin response (I Sec_0-12_, I Sec_12-12_), disposition index {DI} (measured with hyperglycemic clamp) and WBISI using pre and post Rx OGTT. The investigators did not find any improvement I Sec_0-12_, I Sec_12-120_ results which are in line with our results which showed no improvements in the indices derived either from the clamp or OGTT. The difference between this study in adults and ours in adolescents is that their vitamin D levels rose from 17.2 ng/mL (43 mmol/L) at baseline to 34 ng/mL at the end of the 8 week study (29). The fact that they normalized the vitamin D levels supports their findings that vitamin D given in high doses over a short period does not improve metabolic profile of prediabetes and T2DM adults.

To further analyze the data we stratified and looked at the associations between vitamin D levels and metabolic parameters which reflect emerging decompensation such as: Glucose ≥ 100 mg/dL or ≥ 140 mg/dL at 0 and 120 minutes of the OGTT, HbA1c ≥ 5.7%-6.4% (defined as prediabetes by American Diabetes Association). No differences were found in the Rx group using this stratification analysis. No significant differences between treatment and placebo at other time point of OGTTs were found, as well as the mean values of all time points (10, 30, 60, and 90 min) in both glucose and insulin levels. These results further reiterate that the intervention had no effects in the variables of interest.

The cross-over of our RCT was designed to balance the exposure to vitamin D and placebo in sequence based on the arm that the adolescents were assigned to. Each patient served as their own control which allowed for a smaller sample size. The limitations were the “order” and “carry over” effect of a cross over study and we recognize that we did not have a wash out period which could have affected our results. A strength of the study was that we did adjust for seasonal variation in our analysis of the data. We were not able to normalize 25 OHD level and that is a major limitation of our study. 25 OHD has a threshold effect on the beta cell function and we speculate that this is reason why we could find any improvements in insulin secretory and sensitivity parameters. Also, given emerging information on pharmacokinetics studies on available formulations found that ergocalciferol was not as good a choice as cholecalciferol which is more effective in increasing the serum 25(OH) D pools (30).

Our results are aligned to the negative results found in the recent studies showing no beneficial effect of vitamin D on glucose and insulin indices derived from OGTT. We suggest considering much higher dosing for obese adolescents based on adult studies (31) accepting the fact that these are adult sized adolescents. Based on our study there is no evidence to support the use of high dose vitamin D over a short term period to improve glucose homeostasis.
